# Accelerated age-hardening kinetics in additively manufactured 18Ni(300) maraging steel

**DOI:** 10.1038/s41598-025-19047-x

**Published:** 2025-10-08

**Authors:** Shahrzad Sajjadivand, Mark Hartnett, Lu Yang, Wajira Mirihanage, Mert Celikin

**Affiliations:** 1 School of Mechanical and Materials Engineering, UCD, Dublin, D04 V1W8 Ireland; 2 I-Form, the SFI Research Centre for Advanced Manufacturing, UCD, Dublin, D04 V1W8 Ireland; 3 Irish Manufacturing Research, Mullingar, N91 TX80 Ireland; 4 Department of Materials Engineering, The University of Manchester, Manchester, M13 9PL UK; 5 Conway Institute, UCD, Dublin, Ireland

**Keywords:** Maraging steel, Laser-based powder bed fusion (L-PBF), Age-hardening, Electron Back-Scatter diffraction (EBSD), Metals and alloys, Mechanical properties, Design, synthesis and processing, Characterization and analytical techniques, Imaging techniques, Scanning electron microscopy, Engineering, Materials science

## Abstract

In this comparative study between Laser Powder Bed Fusion (L-PBF) versus conventional hot-rolled 18Ni(300) maraging steel, accelerated age-hardening kinetics was determined due to the variation in annealing behaviour. Conventionally produced 18Ni(300) maraging steel exhibited significant grain growth upon solution annealing at 850–1050 °C, which was not evident in the L-PBF counterpart where oxide particles present in as-built condition inhibited grain growth. Electron Backscatter Diffraction (EBSD) analysis showed higher thermal stability of additively manufactured components up to 1050 °C, where significant grain growth was found in hot-rolled and annealed parts. Despite these differences, both processing routes achieved similar peak hardness after ageing, although L-PBF samples displayed faster initial hardening due to enhanced precipitation kinetics linked to finer grain structure and higher grain boundary density.

## Introduction

In recent years, additive manufacturing (AM) has become an emerging technology for the production of critical components used in the aerospace, biomedical, and tooling industries. AM offers several advantages, such as the ability to produce complex geometries, eliminating the need for expensive tooling, reducing assembly requirements, and enabling significant weight reduction and customisation^1,2^. Laser-based powder bed fusion (L-PBF) is one of the most common metal AM techniques. In this process, fine metallic particles are fused by a high-energy laser moving at a high scanning speed to create 3D parts layer by layer with complex geometries that are challenging or impossible to achieve with conventional methods while being cost-effective compared to conventional processes^3,4^. In addition, it is possible to visualise the design in advance as a proof of concept and to make modifications before fabrication^5,6^. The key requirement for AM components is to possess mechanical properties comparable to conventionally produced parts^7^. However, L-PBF-produced parts are typically characterised by high levels of porosity, metastable microstructure, and texture that deteriorate their mechanical performance, thereby limiting the use of AM in the industry^8,9^.

18Ni(300) maraging steels are low-carbon, ultra-high-strength steels belonging to an exclusive class widely used in aerospace, automotive, and tool/die industries. High strength, toughness, and superior weldability along with dimensional stability make maraging steels indispensable^10^. Maraging refers to the martensitic microstructure common to steel, which can be strengthened through age-hardening. Maraging steel consists of a low-carbon martensitic matrix in which ultra-high strength is achieved through the precipitation of intermetallic compounds. The matrix is typically free of interstitial alloying elements due to very low carbon content which not only makes maraging steels excellent candidates for L-PBF based manufacturing but also eliminates the implementation of measures to prevent carbide formation^11,12^.

Conventional 18Ni(300) maraging steels undergo a two-step heat treatment involving solution annealing and age-hardening. Solution annealing involves heating the alloy above the austenite finish temperature (A_f_) to dissolve the secondary phases, while age-hardening treatment allows the formation of Fe, Ni, Ti, Mo and Al-based precipitates^13^. Precipitates with acicular morphology in peak-aged conditions were reported as Ni_3_Ti/Ni_3_Mo phases, whereas the spherical precipitates in over-aged conditions were defined as Laves-Fe_2_Mo or Fe_7_Mo_6_ phases. The maraging steels produced by L-PBF have finer microstructure containing more retained austenite due to rapid cooling (10^6^ ∼ 10^8^ K/s)^14^. A higher level of retained austenite is present due to chemical inhomogeneity caused by microsegregation during solidification. Accordingly, different starting microstructures lead to variations in mechanical properties after post-processing^15,16^.

Several studies have been conducted on L-PBF 18Ni(300) maraging steel to improve mechanical properties similar to conventional ones by optimising the post-processing heat treatment^6,11,12,17–20^. The most common heat treatment route for conventional 18Ni(300) maraging steels is solution annealing at 815 °C for 1 h, followed by aging at 455–510 °C for 3 to 12 h^21,22^. In recent studies, it has been found that there is considerable variation in published data regarding the recommended post-processing heat treatment for L-PBF 18Ni maraging steel. LPBF-built maraging steel is best treated with a combined solution-aging process^19,20,23,24^. Karlapudy et al.^25^ observed that solution annealing followed by dual aging resulted in a more uniform microstructure and reduced anisotropy. According to Bai et al.^11^solution annealing at 900 °C for 1 h and aging at 520 °C for 6 h improved the mechanical performance of maraging steel fabricated by L-PBF. However, the inherent variability in AM processes may prevent the parameters from being universally applicable. Additionally, Mutua et al.^6^ studied the impact of different post-processing heat treatments and noted that specific combinations of solution annealing and aging were needed to tailor the properties of maraging steel to meet specific application requirements. In contrast, some researchers, such as Jägle et al.^15,26,27^ and Casati et al.^17^believe that direct aging without prior solution annealing may be sufficient for certain applications of L-PBF 18Ni(300) maraging steel. According to these studies, direct aging can simplify the post-processing process without compensating mechanical properties, particularly when the as-built microstructure is adequately controlled during the L-PBF process. Since as-built microstructure of LPBF 18Ni(300) differs significantly not only by variation in processing conditions but also from conventional 18Ni(300) maraging steels^14,28^understanding the relationship between microstructural evolution with post-processing heat treatment is crucial.

This study investigates the microstructural evolution of conventionally processed and L-PBF-produced 18Ni(300) maraging steel during solution annealing and age hardening. The aim is to understand how differences in initial microstructure, distinct to the different processing routes, affect grain growth and age-hardening kinetics when subjected to identical heat treatments. Elucidating these mechanisms enable precise microstructural control and tailoring of mechanical properties in additively manufactured maraging steel. Thus, the insights gained will support the development of optimised heat treatment protocols for enhancing the performance of L-PBF maraging steels across various industrial applications.

## Materials and methods

### Additive manufacturing

**Fig. 1:**
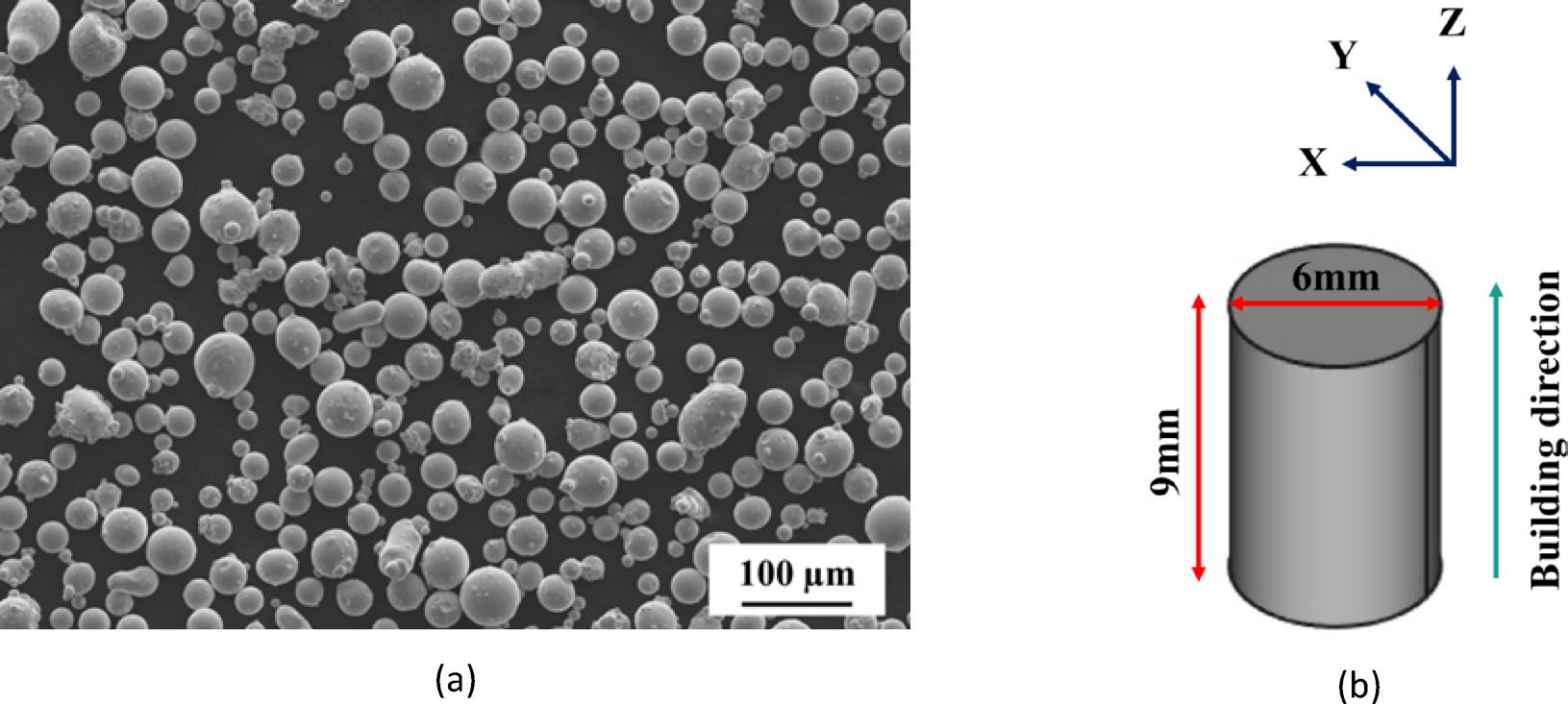
(**a**) Morphology of 18Ni(300) maraging steel powder, (**b**) vertically fabricated L-PBF 18Ni(300) maraging steel cylinders.

18Ni(300) maraging steel powder (SLM Solutions, Lübeck, Germany) with particle sizes of 20–63 μm was used in this study (Fig. 1a). The chemical composition of the 18Ni(300) maraging steel powder is given in Table 1. SLM Solutions - SLM 280 2.0 equipment was used to fabricate cylindrical samples 6 mm in diameter and 9 mm in height (Fig. 1b). All samples were built in the Z + direction following ISO/ASTM 52,921, using a meander scanning strategy with 67° rotation between successive layers. The cylinders were produced using the optimised process parameters listed in Table 2. High-purity argon (Ar) was used to fill the processing chamber during printing. As a baseline, conventional (hot-rolled - rough turned) 18Ni (300) maraging steel bar was purchased in a solution annealed state. The as-received bar was machined into cylinders of 6 ± 0.1 mm diameter and 9 ± 0.1 mm height.

**Table 1:** Chemical composition of 18Ni(300) maraging steel (wt%) used to print the cylinders.

Elements	Fe	Ni	Co	Mo	Ti	Al	Mn	Si	C	*P*	S
Min.	Bal.	18.00	8.50	4.70	0.50	0.05					
Max.		19.00	9.50	5.20	0.80	0.15	0.10	0.10	0.03	0.01	0.01

**Table 2:** Optimised process parameters of L-PBF.

Laser power (w)	Scanning speed (mm/s)	Layer thickness (µm)	Hatch distance (µm)
200	800	30	120

### Heat treatment

L-PBF samples and conventionally produced parts were heat treated in a tube furnace (Nabertherm, Lilienthal, Germany) using a controlled Ar atmosphere (99.98% purity). Specimens were solution annealed for 1 h at three (3) different temperatures of 850 °C, 950 °C, and 1050 °C. The conventionally purchased 18Ni(300) maraging steel was initially solution annealed at a temperature of 816 °C for one hour. However, this temperature was lower than our target temperatures for comparable studies with L-PBF samples. Consequently, these samples were solution annealed again to ensure consistency with the conditions applied to the L-PBF samples. The solution annealed samples at 850 °C for 1 h were aged 45 min to 12 h at a temperature range from 425 to 540 °C, as conditions are listed in Table 3. All the samples were heated using a ramp rate of 10 °C/min followed by air cooling.

**Table 3:** Aging conditions applied for both L-PBF and conventional 18Ni(300) maraging steel samples.

Temperature (°C)	Time (h)
425	6
540	6
490	0.75
490	1.5
490	2
490	3
490	4.5
490	6
490	12

All samples are in the solution annealed state (850 °C–1 h).

### Microstructural characterisation

For microstructural analysis, L-PBF specimens in as-built and heat-treated conditions, as well as conventionally produced specimens after heat treatment, were ground with 320 grit SiC paper. Following this, all samples were polished with diamond suspensions of 9 μm, 3 μm, and 1 μm, and then polished with silica suspension of 0.04 μm. Etching was done using 9.5% nital reagent (nitric acid:15 ml, DI water: 100 ml) for a period of 20 s. Microstructural analysis was performed using a scanning electron microscope (SEM, Hitachi TM4000II, Ibaraki, Japan) at a voltage of 15 kV with a working distance of approximately 6 mm. For EBSD analysis, specimens were prepared through standard metallography sample preparation routes that include grinding (320, 600, 1200, and 2500 SiC grit) and polishing (6 μm, 3 μm and 1 μm) and then finished with 0.04 μm colloidal silica solution for an extended duration to achieve the mirror finish surface required for EBSD imaging. EBSD scans were conducted on a Field Emission Scanning Electron Microscope (FE-SEM, Tescan Mira3) equipped with an EBSD detector (Symmetry Detector, Oxford Instruments), and the SEM was operated at 20 kV with a working distance of 15 mm. AZtec 6.0 data acquisition software (Oxford Instruments) was used with a step size of 0.5 μm to achieve the best spatial resolution during EBSD mapping. The EBSD data further processed using Aztec Crystal post-processing software. The EBSD data further processed using Aztec Crystal post-processing software. For prior austenite grain reconstruction, Kurdjumov–Sachs (K–S) orientation relationship was used as implemented in AztecCrystal (Oxford Instruments). A misorientation threshold of 5° was applied to group martensitic variants belonging to the same parent austenite grain. A minimum grain size of 5 pixels was used to filter out noise and artefacts.

X-ray Diffraction (XRD) analysis was conducted on both as-built and heat-treated samples using a Siemens D500 X-ray diffractometer equipped with a Cu-Kα source (wavelength: 1.5406 Å). The measurements were taken over a 20° to 85° range, with a step size of 0.02° and a holding time of 2s/step. The peak position and full-width half maximum (FWHM) were determined using OriginPro 2024b, nonlinear curve fit (Gauss). For the Williamson–Hall analysis, all distinguishable diffraction peaks corresponding to the martensitic phase, specifically (110), (200), and (211) were used. Background correction was performed implicitly by the Gauss multi-peak fitting function in OriginPro, which accounted for baseline intensity during curve fitting. Semi-quantitative phase fractions were calculated using the Match! Software based on peak intensities. Based on XRD data, the Uniform Deformation Model, a modified version of the Williamson-Hall method (Eq. 1)^29,30^ was used to calculate the microstrain. This approach involves plotting β* cosθ for each measured peak, where *β* is the full width at half maximum (FWHM) and *θ* is the Bragg angle. The slope of the resulting line provides an estimate of the microstrain in the material.

1 $$\:\beta\:*\text{c}\text{o}\text{s}\left(\theta\:\right)=\:k\lambda\:/\left(size\right)+\left(strain\right)*\text{s}\text{i}\text{n}\left(\theta\:\right)$$

### Mechanical testing

The Vickers hardness testing was performed on all specimens in as-built and heat-treated conditions. Similarly, hardness testing was done for conventionally produced samples upon heat treatment. The hardness test was carried out by Mitutoyo hardness tester, AVK C-2 series, under the load of 10 kg-force (kgf) for 20s. To flatten the bases of the cylindrical samples before hardness measurements, all samples were ground with 320, 600, and 1200 grit SiC paper. Each cylindrical sample was subjected to a hardness measurement at its base, i.e. *xy*-plane perpendicular to build direction (z). Hardness testing was conducted for each sample with 7 repetitions and the standard deviation was calculated.

## Results and discussion

### XRD analysis

**Fig. 2:**
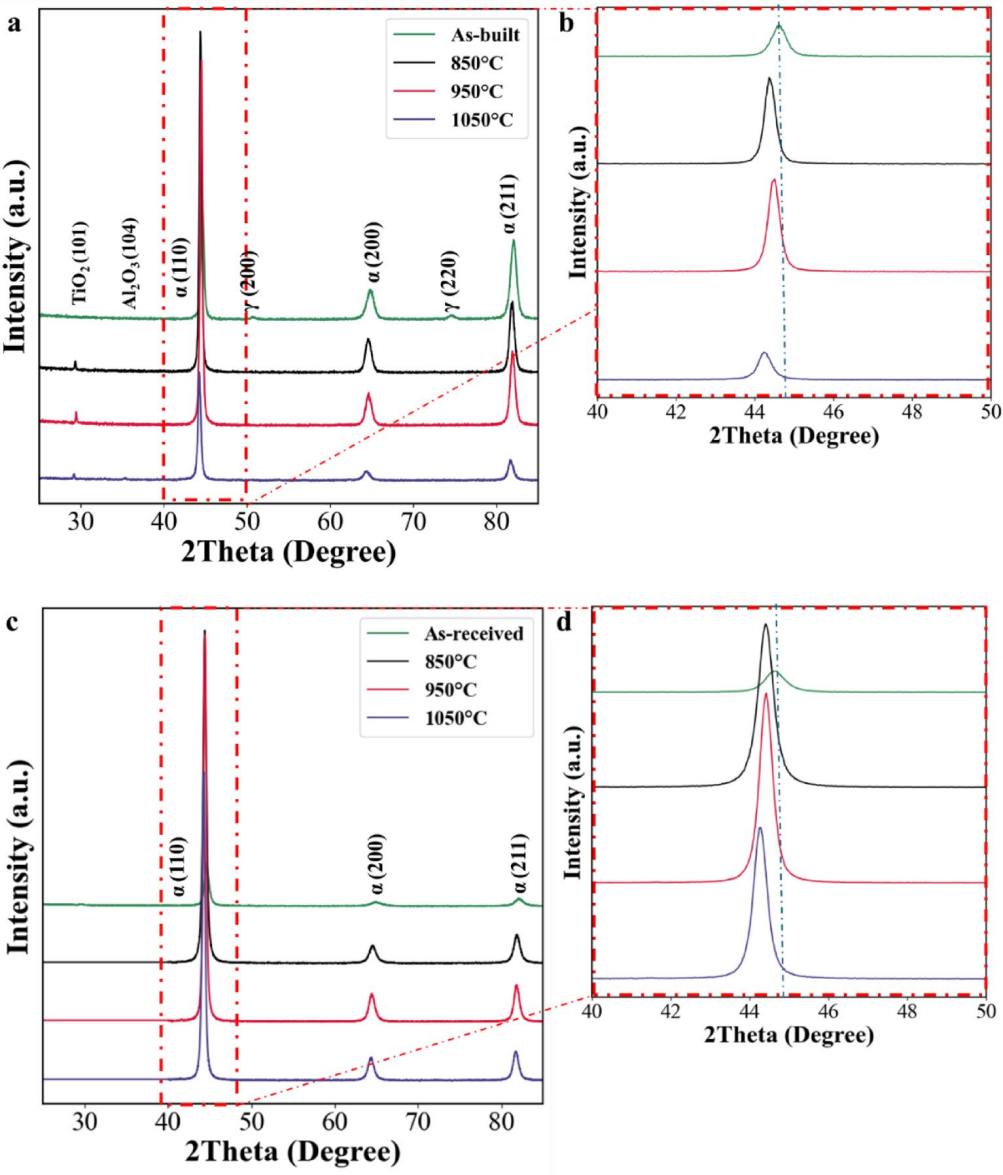
XRD patterns of (**a**,**c**) L-PBF and conventional 18Ni(300) maraging steel in as-built and solution annealed conditions; (**b**,**d**) L-PBF and conventional 18Ni(300) maraging steel in as-built and solution annealed conditions (2θ = 40–50°).

Figure 2a shows the XRD patterns of as-built and solution annealed L-PBF 18Ni(300) maraging steel within the 2Theta range of 20°- 85°. Martensite (α-phase) and retained austenite (γ-phase) were present in as-built condition, confirming that austenite was almost transformed into martensite during the L-PBF process, however, a small amount of austenite was retained. By solution annealing the L-PBF samples at various temperatures for 1 h, the peaks related to retained austenite, (200)_γ_ and (220)_γ_, disappeared. Therefore, it can be stated that austenite was transformed into martensite after solution annealing up to 1050 °C, however it should be note that the matrix might still contain austenite phase which cannot be detected by XRD due to resolution limit. The intensity of the main martensite peak (110)_α_ is higher for the samples solution annealed at 850 °C and 950 °C compared to as-built (Fig. 2b). This observation can be attributed to almost fully transformation of austenite to martensite and development of related texture.

In all solution annealing conditions, a new peak corresponding to TiO_2_, indexed as (101)_TiO2_, was observed. Initially detected at 850 °C, the intensity of this peak slightly increased at 950 °C, potentially indicating either the coarsening and / or the increase in the number of TiO_2_ particles with rising temperature. A new peak corresponding to the (104)_Al2O3_ plane of Al_2_O_3_ emerged at 1050 °C, indicating the formation of a new oxide phase. As the temperature increases, the solubility of Ti in the matrix also increases, reducing its tendency to react with oxygen, thereby allowing oxygen to preferentially react with Al, leading to the formation of Al_2_O_3_ at higher temperatures. To support these qualitative observations, Table 4 presents the estimated volume fractions of TiO_2_, Al_2_O_3_, and retained austenite. These values provide a quantitative basis for understanding oxide evolution and austenite dissolution, which influence grain boundary stability and transformation behaviour during solution annealing. Based on Riabov et al.^31^ and Deng et al.^32^ oxide particles are typically in nanoscale and tend to form during processing through reactions between oxygen and strong oxygen-affinitive elements such as Ti, Al, and Si a and are known to form uniformly dispersed inclusions within the matrix during L-PBF processing. These oxides can play a dual role in microstructure evolution. Uniformly dispersed, fine oxides can effectively impede grain boundary motion through the Zener pinning mechanism, thereby restricting grain growth during heat treatment. In addition, they may contribute to strengthening by hindering dislocation movement through Orowan looping mechanisms^31^. However, coarse or irregular inclusions located at grain boundaries can serve as stress concentrators, potentially compromising ductility and fracture resistance. This detrimental effect has been observed in other L-PBF alloys containing oxide inclusions enriched in elements such as silicon and manganese^33^.

Peak positions of all solution-annealed samples are shifted to lower 2θ angles compared to the as-built condition (Fig. 2b), indicating lattice expansion due to lattice relaxation. In the L-PBF samples, the formation of oxides such as Al_2_O_3_ and TiO_2_ suggests that larger atoms like Ti (1.47 Å) and Al (1.43 Å) are removed from the matrix. Their removal reduces lattice distortion in austenite, leading to relaxed martensitic lattice upon cooling. Additionally, Solution annealing facilitates the thermally activated movement and annihilation of dislocations, leading to a reduction in dislocation density and associated lattice strain. Together, these effects result in the observed peak shift toward lower angles. In Fig. 2c, the XRD pattern is shown for conventional 18Ni(300) maraging steel that has been solution annealed using the same temperature range. These samples were initially hot-rolled, a process known to introduce high dislocation density in the material. However, during solution annealing at 816 °C, the deformation-induced microstructure reverts to austenite, and most of the dislocations associated with hot-rolling is removed. Upon cooling, a fully martensitic structure forms at room temperature in both the as-received and solution-annealed samples, with no evidence of retained austenite. As shown in Fig. 2d, peak positions shift to lower 2θ values with increasing solution annealing temperature compared to the as-received condition. This shift is attributed to further stress relief and reduction in dislocation density during annealing, which leads to lattice expansion, as supported by the data in Table 5. Notably, no oxide phases were detected in the conventionally processed samples. This absence, likely due to the lack of powder-derived surface oxides and the more controlled thermal and chemical environment, may facilitate more effective stress relief during heat treatment compared to the L-PBF counterparts.

**Table 4:** Quantitative phase fractions (vol%) of martensite, retained austenite, TiO_2_, and Al_2_O_3_ in L-PBF 18Ni(300) maraging steel in as-built and different solution annealed conditions.

	Volume fraction (%)			
	Martensite	Retained austenite	TiO_2_	Al_2_O_3_
As-build	95.91	4.09	0.00	0.00
850 °C–1 h	95.64	0.00	4.36	0.00
950 °C–1 h	93.97	0.00	6.03	0.00
1050 °C–1 h	80.57	0.00	14.46	4.97

Table 5 summarises the quantitative analysis of the (110)α peak, including 2θ position, calculated lattice parameter, and FWHM, for all conditions. These values support the previously discussed trends of lattice expansion and structural relaxation upon solution treatment. In particular, the FWHM data provide additional insight into microstructural evolution, reflecting the progression from a strained, dislocation-rich structure in the as-built condition to a more recovered state at higher annealing temperatures. The quantitative data complement the XRD pattern shifts shown in Fig. 2 and reinforce the interpretation of stress relief and compositional homogenisation, and transformation-induced lattice distortion during heat treatment.

**Table 5:** Calculated peak positions, lattice constants, and FWHM for martensite phase (110) plane of L-PBF and conventional 18Ni(300) maraging steel at different conditions.

	L-PBF			Conventional			
	2θ (°)	Lattice constant (Å)	FWHM (Radian)		2θ (°)	Lattice constant (Å)	FWHM (Radian)
As-build	44.61	2.8702	0.0075	As-received	44.66	2.8672	0.0103
850 °C–1 h	44.26	2.8915	0.0054	850 °C–1 h	44.38	2.8844	0.0078
950 °C–1 h	44.48	2.8782	0.0057	950 °C–1 h	44.48	2.8782	0.0066
1050 °C–1 h	44.25	2.8924	0.0067	1050 °C–1 h	44.25	2.8924	0.0068

Full calculation details are available in the Supplementary Information.

**Fig. 3:**
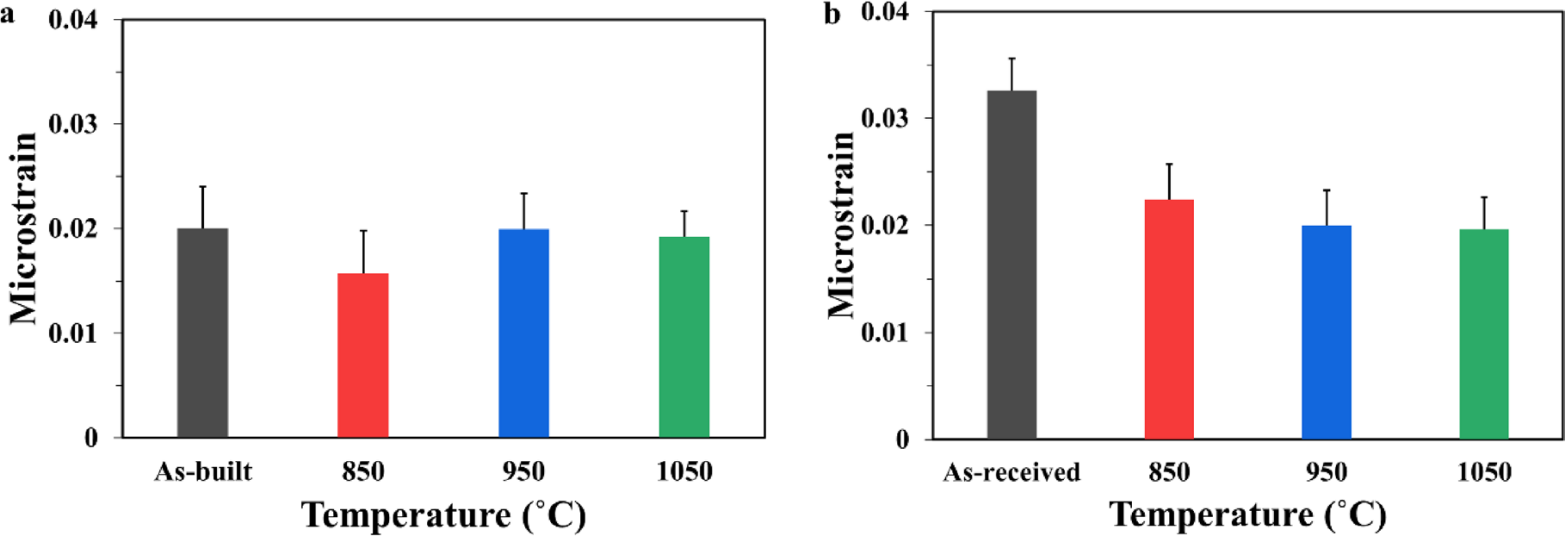
Microstrain analysis of 18Ni(300) maraging steel at different solution annealed conditions for (**a**) L-PBF and (**b**) conventional.

Microstrain was determined using the Uniform Deformation Model, a modified version of the Williamson-Hall method^29,34^ based on the XRD data (Fig. 2) that is presented in Fig. 3 for both L-PBF and conventional 18Ni(300) maraging steel. In the L-PBF samples (Fig. 3a), microstrain results show statistically no significant change in microstrain between the as-built and solution-annealed conditions. There is a slight drop after annealing at 850 °C, followed by a small increase at higher temperatures (950 °C and 1050 °C). The reduction after 850 °C is likely due to the formation of TiO_2_ and Al_2_O_3_ oxides, which remove large solute atoms like Ti and Al from the matrix. Their removal decreases lattice distortion caused by size mismatch with smaller Fe atoms, leading to lower strain in the austenite and, consequently, in the martensite formed during cooling. The increase in microstrain at higher temperatures may be due to a larger temperature difference (ΔT) during cooling, which enhances transformation-induced stress. In all cases, the microstrain results from a combination of oxide formation and stress generated during martensitic transformation. On the other hand, in the conventional samples, microstrain decreases slightly from 0.0326 in the as-received state (solution annealed at 816 °C) to 0.0224, 0.0200, and 0.0196 at 850 °C, 950 °C, and 1050 °C, respectively (Fig. 3b). In the as-received condition, microstrain is primarily attributed to the martensitic transformation during cooling, which introduces internal lattice distortion. A small contribution from residual deformation-induced dislocations introduced during prior hot-rolling may also be present. However, after solution annealing at 850 °C and above, the microstrain mainly comes from the martensitic transformation, as deformation-related stress has likely been removed.

There was good agreement between the phases determined via XRD analysis of L-PBF and conventional 18Ni(300) maraging steel and those reported previously^6,14,35–38^. In the as-built L-PBF samples, martensite peaks are predominant, which aligns with the rapid cooling rates of the L-PBF process that favour martensitic transformation over austenite retention. However, a small fraction of retained austenite persists, suggesting incomplete transformation, potentially due to local variations in cooling rates or chemical heterogeneity. Krol et al.^39^ reported that the microsegregation of elements like Ni can stabilize the austenite phase, hindering its complete transformation into martensite in as-built L-PBF 18Ni(300) maraging steel^36,40^. The transformed martensite peak shift in L-PBF solution annealed samples at 850°Cand 950 °C, and 1050 °C compared to as-built state as shown in Fig. 2b, aligns with the observed microstrain change in these samples, as depicted in Fig. 3a.

Formation of oxide particles (Fig. 2a) are typical of L-PBF steels, specially 316 L stainless steels and maraging steels. In L-PBF of steel, oxide particles predominantly arise from the oxygen-rich environment of the feedstock powder, produced via inert gas atomisation. Elements such as Ti and Al within the maraging steel, known for their high oxygen affinity, react with this oxygen during the rapid melting and solidification process of L-PBF. This reaction results in the formation of stable oxides that get trapped within the steel matrix as the melt pool quickly solidifies, forming oxide particles^32,41–43^. It is possible that these oxide particles were present in the as-built condition as well, but in quantities too low to be detected by XRD.

### Microstructural characterisation

**Fig. 4:**
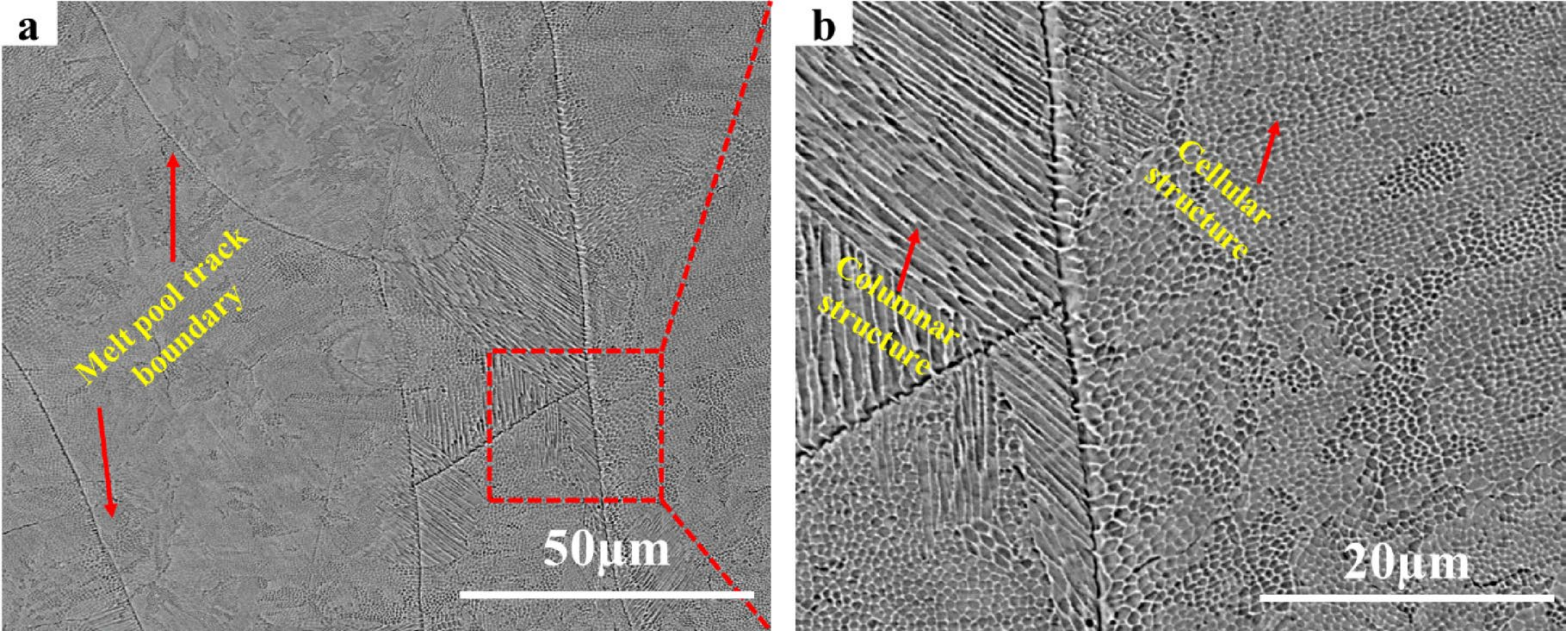
Microstructure of as-built L-PBF 18Ni(300) maraging steel; (**a**) low magnification, (**b**) high magnification.

Figure 4 presents SEM images of as-built L-PBF 18Ni(300) maraging steel indicating cellular/columnar structure and melt pool track boundaries. Fine cellular/columnar microstructure is typical of L-PBF 18Ni(300) maraging steel^11,14^. This type of microstructure is also observed in other alloy systems, such as 316 L stainless steel^44,45^Co-28Cr-6Mo alloy^46^Inconel 625^47^, and Al-Si alloys^48^. Columnar structures are mainly distributed near the melt pool boundary, and massive cellular structures are visible in the centre of the laser track. Microstructural differences are caused by different melt pool temperature gradients. From the Gaussian distribution of laser energy, it can be demonstrated that a temperature gradient forms from the centre of the melt pool with the highest temperature to its edge^49–51^. Consequently, this temperature gradient leads martensite to grow towards the melt pool boundaries and form columnar structures. Cellular structures occur as a result of rapid solidification with a high cooling rate inhibiting crystal growth^11,14^.

**Fig. 5:**
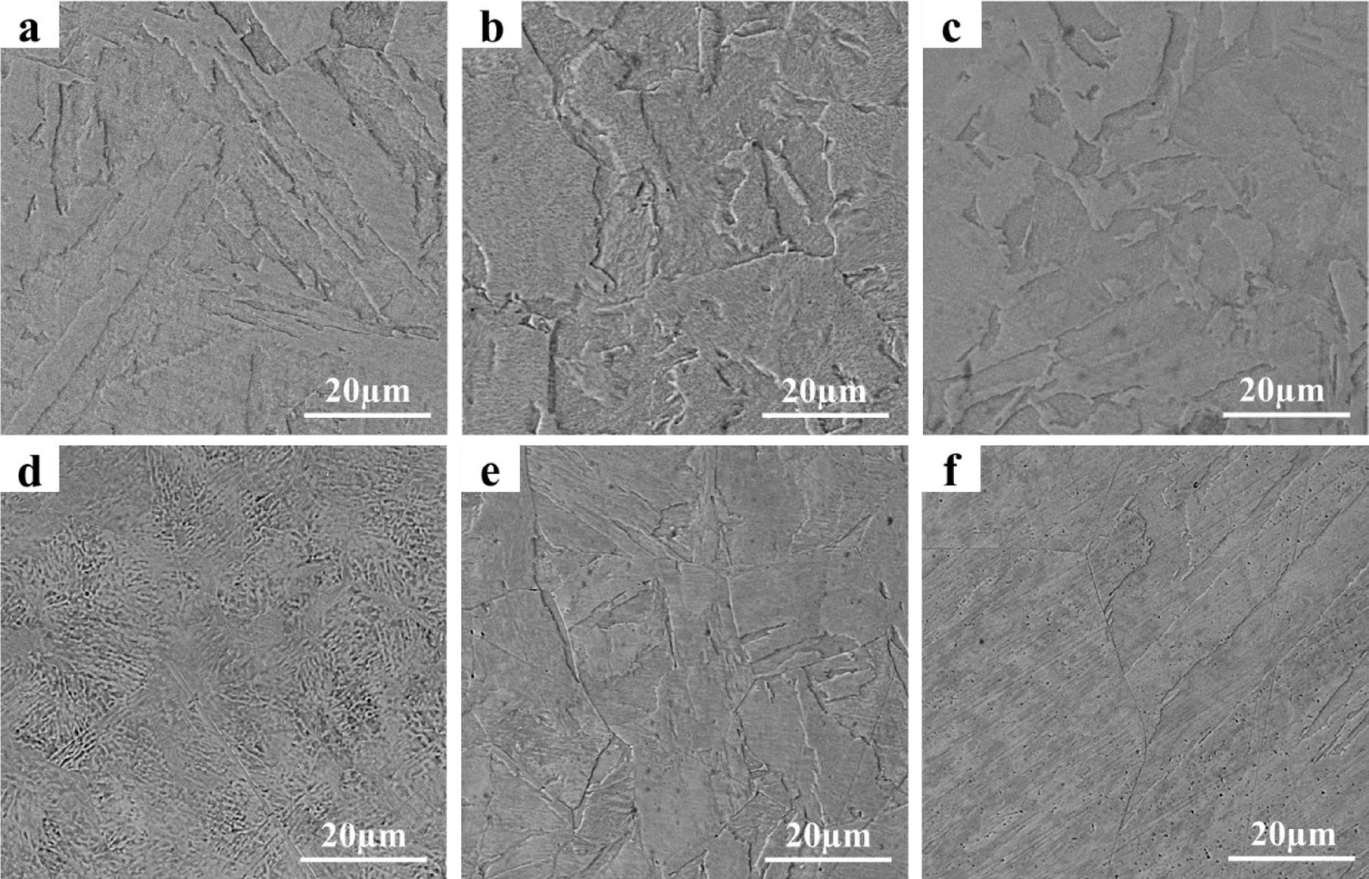
Microstructure of L-PBF 18Ni(300) maraging steel in solution annealed at (**a**) 850 °C–1 h, (**b**) 950 °C–1 h, and (**c**) 1050 °C–1 h condition; also microstructure of conventional 18Ni(300) maraging steel in solution annealed at (**d**) 850 °C–1 h, (**e**) 950 °C–1 h, and (**f**) 1050 °C–1 h state.

Figure 5 shows the microstructure of both L-PBF and conventional 18Ni(300) maraging steel after solution annealing at 850 °C, 950 °C, and 1050 °C for 1 h. After solution annealing at different temperatures in L-PBF samples there has been a clear disappearance of the semi-elliptical shaped boundaries of the melt pools. Moreover, upon solution annealing at 850 °C (Fig. 5a), cellular/columnar martensitic structure is transformed into lath martensite with a coarsened grain structure in L-PBF samples. By increasing the solution annealing temperature up to 950 °C (Fig. 5b) and 1050 °C (Fig. 5c), there is no significant change in the size of the martensite laths compared to 850 °C. At 850 °C, the microstructure of solution annealed L-PBF differs significantly from that of conventional 18Ni(300) maraging steel (Fig. 5d). However, in both processes, the microstructure is observed to be similar when the temperature is increased to 950 °C (Fig. 5b, e). On the other hand, in conventional samples, significant coarsening occurred at 1050 °C (Fig. 5f) compared to the L-PBF. Song et al.^52^Bai et al.^11^and Faiçal et al.^53^ also reported microstructural coarsening and conversion of cellular/columnar martensite to lath martensite upon solution annealing in L-PBF 18Ni(300) maraging steel. The conversion of cellular/columnar to lath martensite during solution annealing is driven by the need to achieve a more equilibrium and lower-energy state. The solution annealing treatment allows the austenite to form more uniformly, and the controlled cooling rate facilitates the formation of lath martensite, which is thermodynamically more stable under these conditions^14^.

**Fig. 6:**
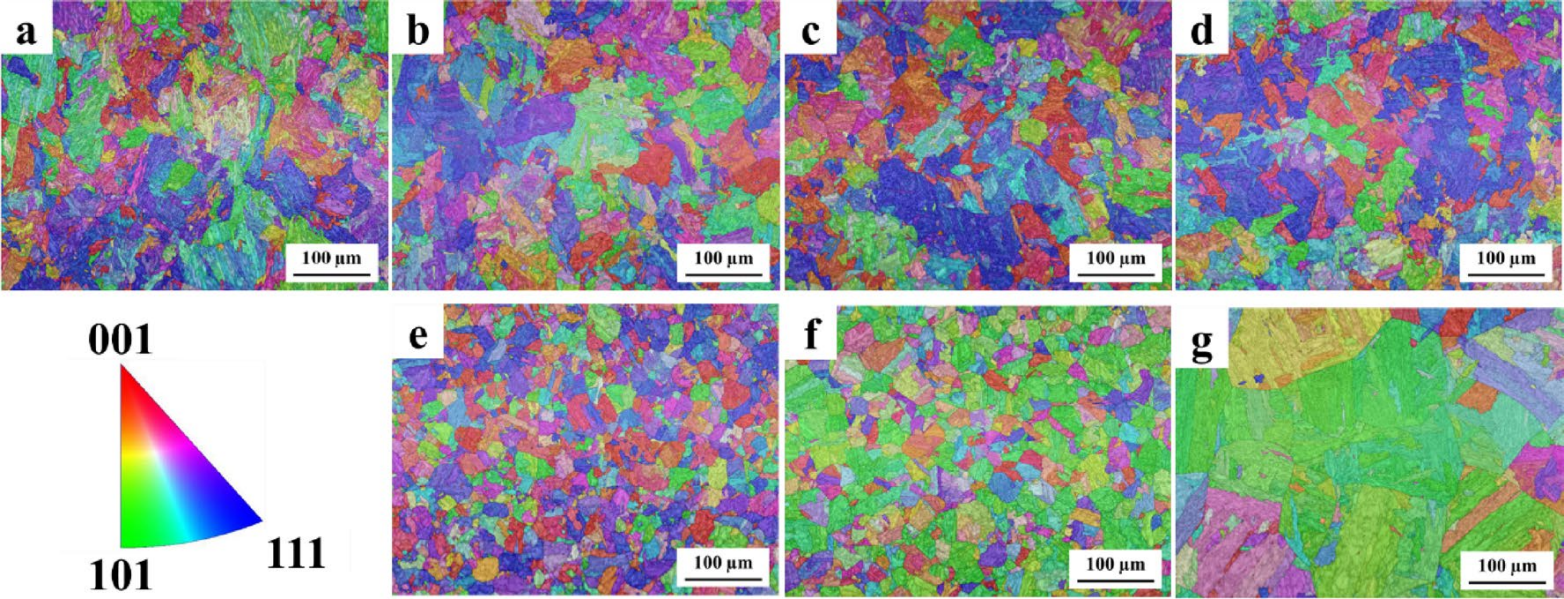
Inverse pole figure (IPF) maps of the prior austenite phase in L-PBF 18Ni(300) maraging steel for: (**a**) as-built condition, and after solution annealing at (**b**) 850 °C–1 h, (**c**) 950 °C–1 h, and (**d**) 1050 °C–1 h. Corresponding microstructures of conventionally produced 18Ni(300) maraging steel after solution annealing at (**e**) 850 °C, (**f**) 950 °C, and (**g**) 1050 °C for 1 h are also shown.

**Fig. 7:**
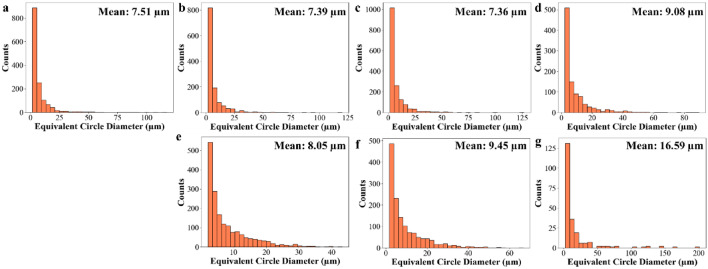
The grain size distribution of prior austenite phase in L-PBF 18Ni(300) maraging steel in (**a**) as-built, (**b**) solution annealed at 850 °C–1 h, (**c**) solution annealed at 950 °C–1 h, and (**d**) solution annealed at 1050 °C–1 h condition; also microstructure of conventional 18Ni(300) maraging steel in (**e**) solution annealed at 850 °C–1 h, (**f**) solution annealed at 950 °C–1 h, and (**g**) solution annealed at 1050 °C–1 h state.

The microstructural evolution with increasing solution annealing temperature was analysed using EBSD for both conventional and L-PBF 18Ni(300) maraging steel samples. To further examine the influence of microstructural characteristic, i.e., oxide particles on martensite formation and transformation behaviour, morphology and size of the prior austenite grains can provide substantial details. As these grains play a critical role in determining the resulting martensitic structure, they critically influence the latter properties such as hardness through precipitation behaviour. Therefore, EBSD data was essential to reconstruct and analyse the prior austenite grain structure under various solution annealing conditions. The inverse pole figures (IPF) of the reconstructed parent austenite phase (Fig. 6) highlights notable microstructural differences between the two processing routes across different solution annealing temperatures. In the as-built L-PBF sample (Fig. 6a), the microstructure consists of small, irregularly shaped prior austenite grains with mean grain size of 7.51 μm (Fig. 7a). After solution annealing at 850 °C for 1 h (Fig. 6b), the grain morphology remains largely irregular, and the average grain size slightly decreases to 7.39 μm (Fig. 7b). At 950 °C (Fig. 6c), the grain shape and distribution appear similar to those at 850 °C, with the average grain size further reducing to 7.36 μm (Fig. 7c). At 1050 °C for 1 h (Fig. 6d), the grains remain fine and heterogeneous, with a moderate increase in average grain size to 9.08 μm (Fig. 7d). Across all conditions, no significant grain coarsening or transition to equiaxed morphology is observed in the L-PBF samples. In contrast, the conventionally produced samples show visibly larger and more distinct prior austenite grains compared to L-PBF counterparts. At 850 °C (Fig. 6e), the grains appear relatively equiaxed and uniform in shape with a mean size of 8.05 μm (Fig. 7e). Upon increasing the annealing temperature to 950 °C (Fig. 6f), the grains grow further, reaching a mean size of 9.45 μm (Fig. 7f). At 1050 °C (Fig. 6g), significant coarsening is evident, with the grain size increasing to 16.59 μm (Fig. 7g).

**Fig. 8:**
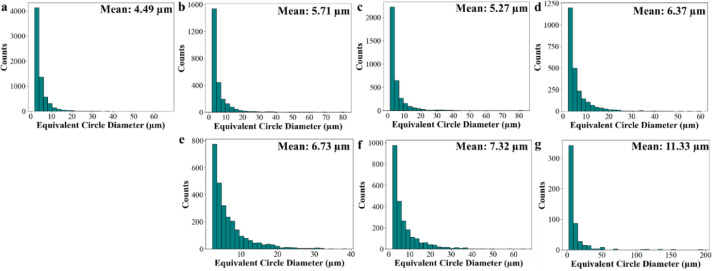
The grain size distribution of martensite phase in L-PBF 18Ni(300) maraging steel in (**a**) as-built, (**b**) solution annealed at 850 °C–1 h, (**c**) solution annealed at 950 °C–1 h, and (**d**) solution annealed at 1050 °C–1 h condition; also microstructure of conventional 18Ni(300) maraging steel in (**e**) solution annealed at 850 °C–1 h, (**f**) solution annealed at 950 °C–1 h, and (**g**) solution annealed at 1050 °C–1 h state.

Figure 8 presents the grain size distribution of the martensitic phase formed after cooling from each solution annealing condition. A clear correlation is observed between the prior austenite grain size (Fig. 7) and the resulting martensitic microstructure. In the L-PBF samples, the mean prior austenite grain size remains relatively fine, ranging from 7.51 μm in the as-built condition to 9.08 μm after solution treatment at 1050 °C. This corresponds to martensite grain sizes between 4.49 μm and 6.37 μm. In comparison, the conventional samples exhibit progressive coarsening of the prior austenite grains, increasing from 8.05 μm at 850 °C to 16.59 μm at 1050 °C. This grain growth is reflected in the martensitic structure, where the average grain size increases from 6.73 μm to 11.33 μm. This relationship shows the crystallographic transformation behaviour between austenite and martensite, which typically follows Kurdjumov–Sachs (K-S) orientation relationships^54,55^. In fine-grained austenite, the increased number of nucleation sites restricts martensite plate growth, resulting in a finer transformed microstructure. In contrast, coarse austenite grains impose fewer constraints, allowing the formation of larger martensite grains. This divergence in thermal response underscore the importance of prior austenite grain size in influencing the size and morphology of the martensitic microstructure, which in turn affects the precipitation kinetics. The inverse pole figure (IPF) maps of the martensitic phase, which support the data shown in Fig. 8, are provided in the Supplementary Information (Fig. S1).

**Fig. 9:**
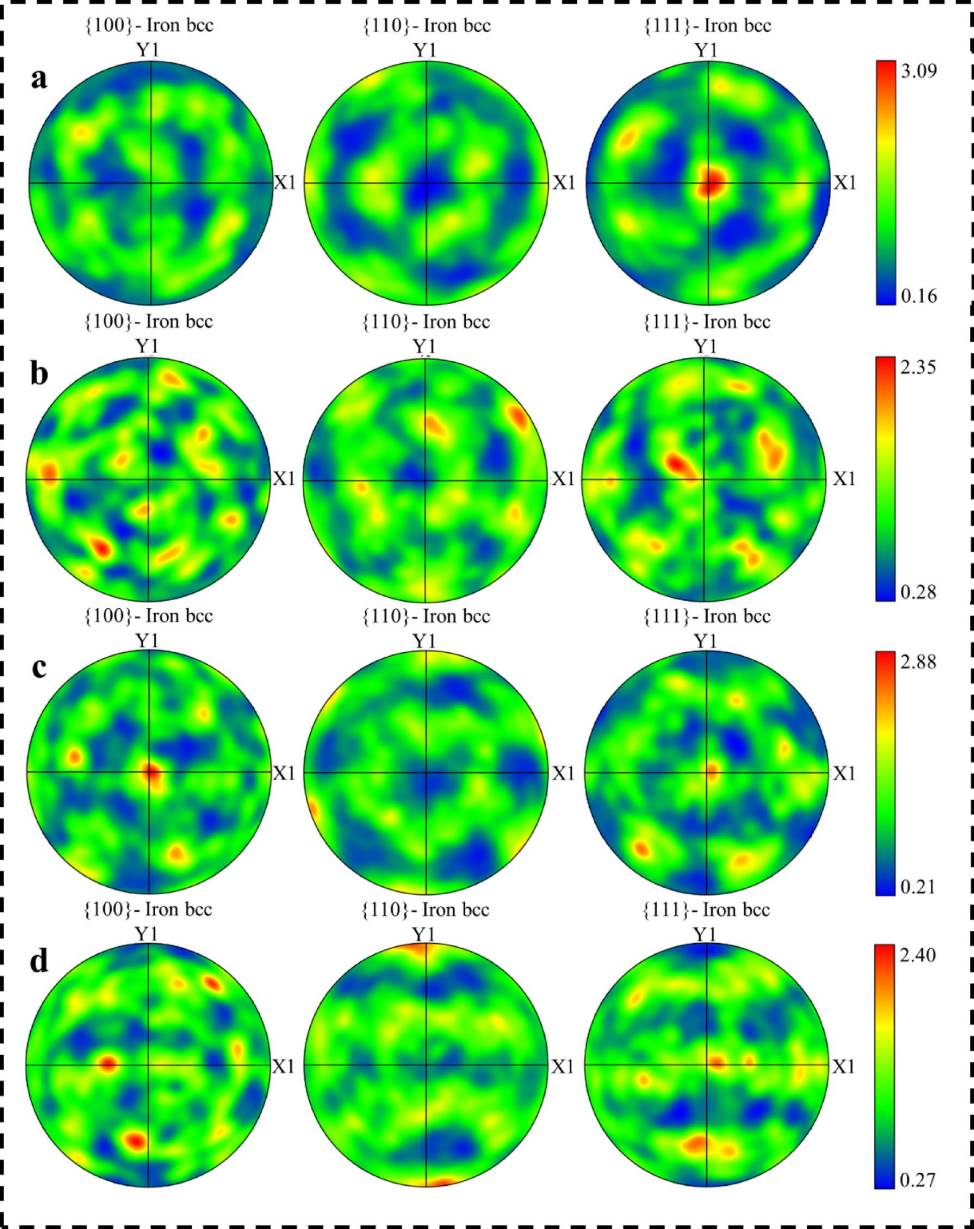

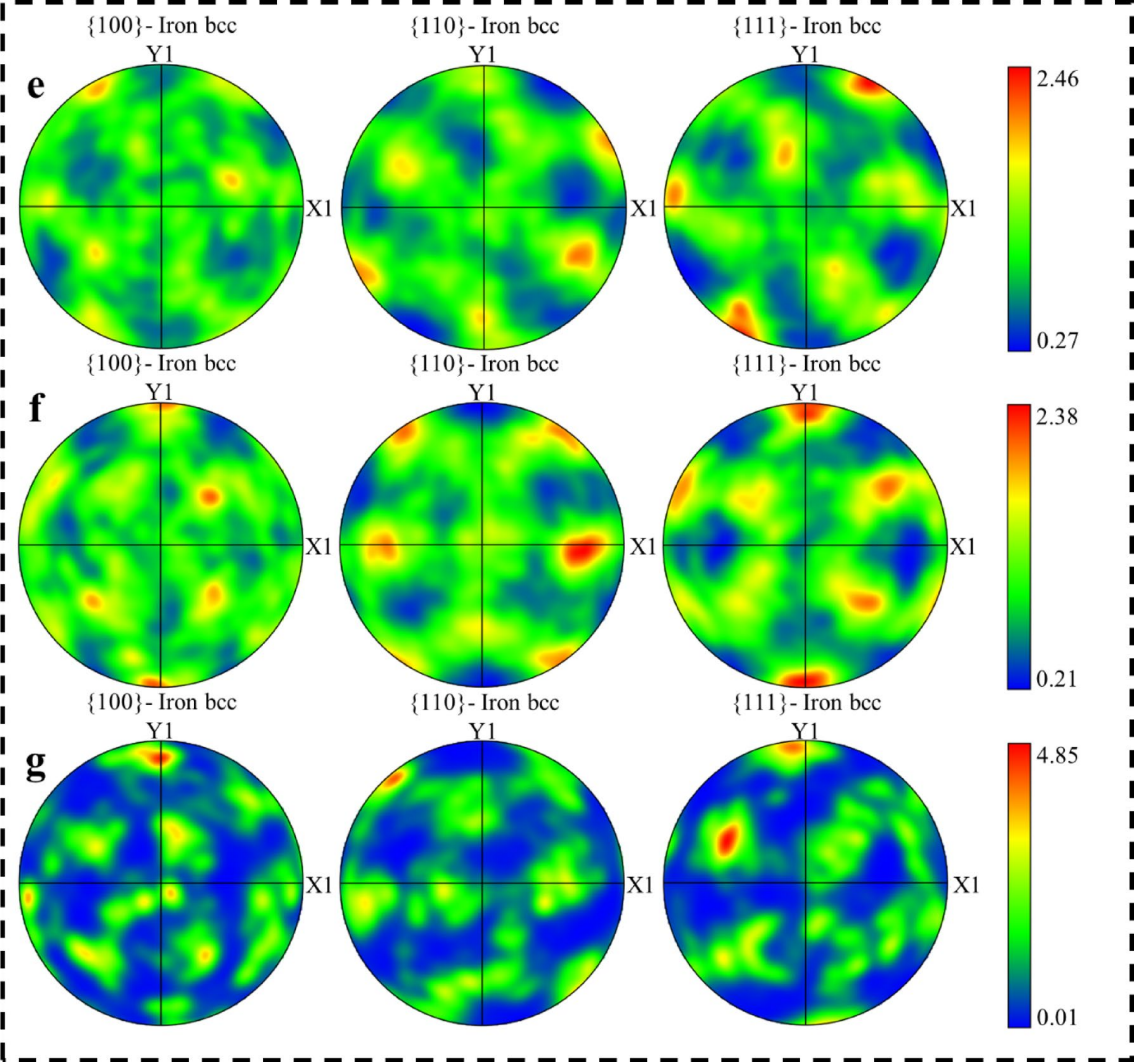
{100}, {110}, and {111} pole figures of the martensite phase in 18Ni(300) maraging steel under various conditions: (**a**) L-PBF as-built condition, and after solution annealing at (**b**) 850 °C–1 h, (**c**) 950 °C–1 h, and 1050 °C-1. Corresponding pole figures of conventionally produced samples solution annealed at (**e**) 850 °C, (**f**) 950 °C, and (**g**) 1050 °C for 1 h are also presented.

The crystallographic texture of the martensitic phase was analysed using EBSD-derived pole figures for both L-PBF and conventional 18Ni(300) maraging steel samples under different solution annealing conditions, as presented in Fig. 9. In the as-built L-PBF sample (Fig. 9a), the pole figures show some variation in texture across the {100}, {110}, and {111} planes, with the {111} plane exhibiting a relatively stronger preferred orientation. Upon solution annealing at increasing temperatures (Fig. 9b–d), the texture in the {111} pole figure becomes more diffuse, indicating a trend toward randomly oriented grains. However, a relative increase in orientation intensity is observed in the {100} pole figure in solution annealed samples compared to as-built, suggesting the development of a weak {100} texture in the L-PBF samples. The texture of the martensitic phase is inherited from the austenite structure present at the solution annealing temperature, as governed by the Kurdjumov–Sachs orientation relationship. In contrast, the conventionally produced samples (Fig. 9e and g) exhibit a gradual increase in texture intensity with increasing solution annealing temperature. The strong intensity observed in Fig. 9g is attributed to the presence of large grains.

**Fig. 10:**
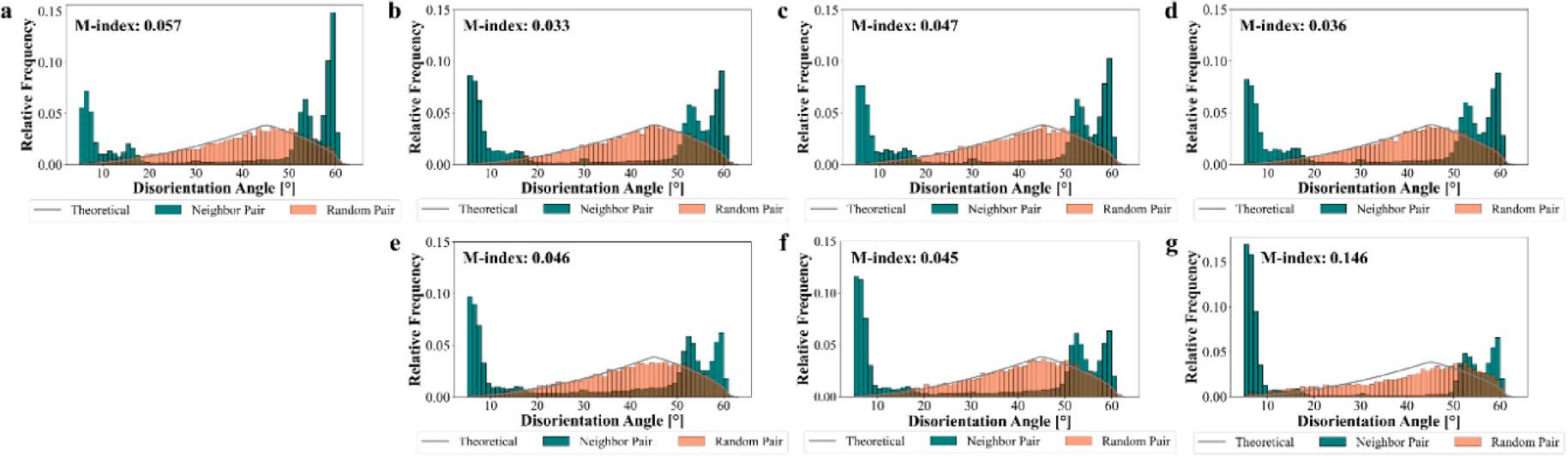
Misorientation angle distribution of L-PBF 18Ni(300) maraging steel in the (**a**) as-built condition, and after solution annealed at (**b**) 850 °C for 1 h, (**c**) 950 °C for 1 h, and (**d**) 1050 °C for 1 h. Corresponding distributions for conventionally processed 18Ni(300) maraging steel are shown for solution treatment at (**e**) 850 °C, (**f**) 950 °C, and (**g**) 1050 °C, each for 1 h.

To further clarify the texture and grain orientation details, Fig. 10 presents the misorientation angle distributions related to different solution annealing conditions, for both L-PBF and conventional 18Ni(300) maraging steel samples. In the L-PBF samples, the fractions of LAGBs remain nearly unchanged at all annealing temperatures. These boundaries correspond to martensite lath boundaries formed during cooling from the austenitic phase. The stable distributions suggest limited grain boundary mobility during annealing, likely due to the pinning effect of oxide particles. The M-index values for the L-PBF samples also remain relatively constant, 0.057 (as-built), 0.033 (850 °C), 0.047 (950 °C), and 0.036 (1050 °C), indicating weak, diffuse texture with no significant evolution during solution treatment. The M-index quantifies the deviation of the measured misorientation distribution from the theoretical Mackenzie distribution for a random texture. Higher values correspond to more pronounced texture, as grains grow and certain orientations become more dominant^56^. Whereas, the conventional samples show a slight increase in the fraction of LAGBs at 950 °C and a more noticeable rise at 1050 °C, which indicates grain growth. Hutchinson et al.^56^ observed similar behaviour in silicon steels, where an increase in LAGBs accompanied extensive grain growth. The M-index in the conventional samples also increases from 0.046 (850 °C) and 0.045 (950 °C) to 0.146 at 1050 °C, suggesting the development of stronger crystallographic texture as shown in Fig. 9 (e-g).

Our findings in maraging steel correspond well with those reported in the literature for AISI 316 L stainless steel. Ronneberg et al.^57^ and Salman et al.^58^ reported that recrystallisation and grain growth kinetics in L-PBF AISI 316 L stainless steel is inactive and starts at higher temperatures than in conventionally-processed counterparts which is due to the in-situ formation presence oxide particles during process. For instance, recrystallisation in 70% cold-rolled AISI 316 L stainless steel is achieved after heating for 2 h at 850 °C, according to one study^59^while another study^60^ found that recrystallisation occurs after 1 h at 1000 °Cin AISI 316 L that has undergone 13% cold-drawing. For specimens that are 95% cold-rolled, recrystallisation begins at 700 °C after 1 h and is complete by 800 °C after another hour^61^. However, for L-PBF AISI 316 L, recrystallisation does not occur until the material is heated to temperatures above 1100 °C^62^.

**Fig. 11:**
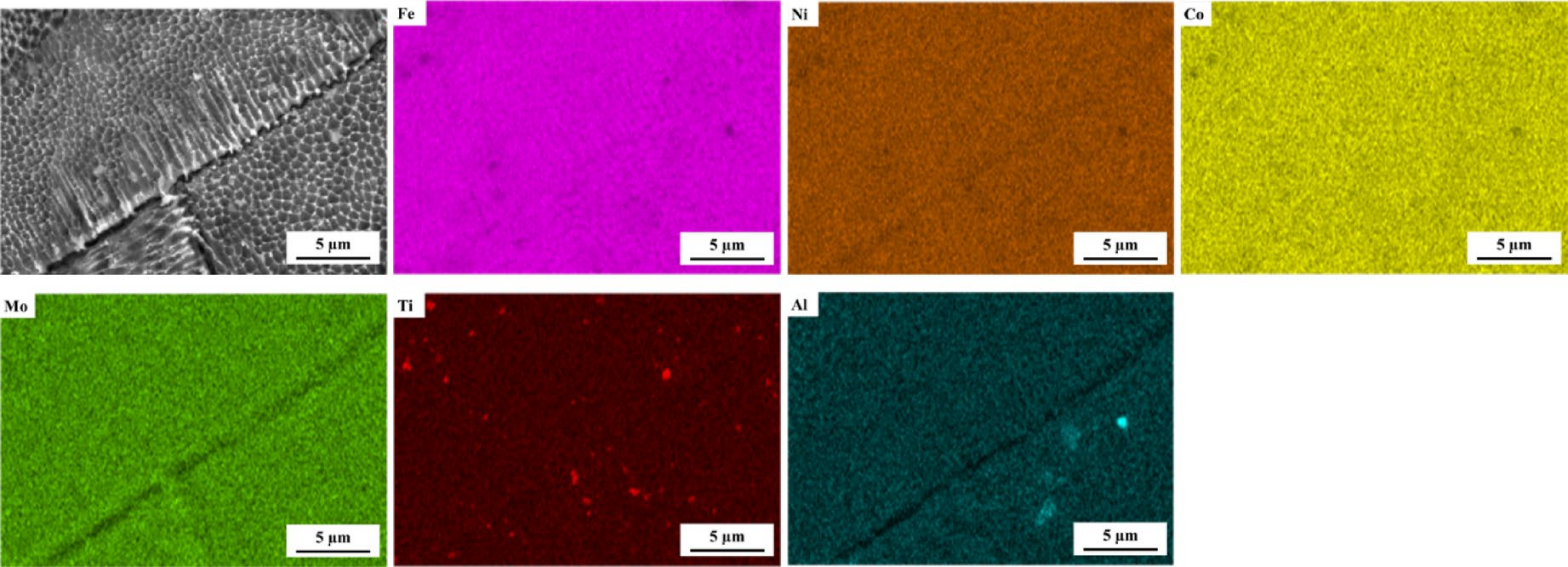
EDS analysis, elemental map of as-built L-PBF 18Ni(300) maraging steel.

Figure 11, EDS analysis, presents the presence of stable Ti-oxide and Al-oxide particles in as-built L-PBF 18Ni(300) maraging steel. Oxide particles dispersed within the L-PBF microstructure exert a pinning pressure on migrating grain boundaries, a phenomenon known as Zener pinning^51,63^. These particles inhibit grain growth by reducing boundary mobility, effectively stabilising the fine microstructure even during solution annealing at elevated temperatures. Zener pinning pressure (Pₓ) is inversely proportional to the particle size and directly proportional to the volume fraction of particles, typically described by:

2 $$\:{P}_{x}\approx\:\left(3\gamma\:f\right)/\left(2r\right)$$

where γ is the grain boundary energy, *f* is the volume fraction, and *r* is the particle radius, the Zener pinning pressure exerted by second-phase particles can effectively inhibit grain boundary migration. As shown in Table 4, the volume fraction of oxide particles (TiO_2_ and Al_2_O_3_) increases progressively with solution annealing temperature in the L-PBF samples. The fine and stable oxide particles observed in Fig. 11 are likely responsible for the pinning effect, as their size and distribution make them effective at hindering grain boundary movement. These oxides are present during the high-temperature annealing stage when the microstructure exists in the austenitic phase, where their interaction with migrating boundaries is most pronounced. As a result, the L-PBF samples exhibit minimal grain boundary movement, which corresponds with the limited grain growth (Fig. 7) and the persistence of LAGBs (Fig. 10) observed in the EBSD data. The presence of oxide particles and their effect on stabilising the high-temperature microstructure are further supported by the XRD data. In addition, porosity, a characteristic feature of the L-PBF process, can similarly restrict grain boundary movement, as it is known that pores can behave like particles in this regard, potentially influencing grain growth and microstructure stability^64^.

In contrast, conventional 18Ni(300) maraging steel, which had undergone hot-rolling and prior solution annealing, entered the current heat treatments potentially in a dynamically recrystallised state. The absence of oxide particles and porosity may have facilitated grain growth during subsequent annealing. The observed progression from equiaxed grains at 850 °C and 950 °C to coarser grains at 1050 °C suggests that grain boundary mobility was less restricted compared to the L-PBF samples. This contrast in thermal response illustrates the important influence of microstructural features such as oxide particles and porosity on the annealing behaviour of L-PBF maraging steel, and emphasises the need to tailor heat treatment strategies according to the specific characteristics introduced by different manufacturing routes.

### Mechanical properties

**Fig. 12:**
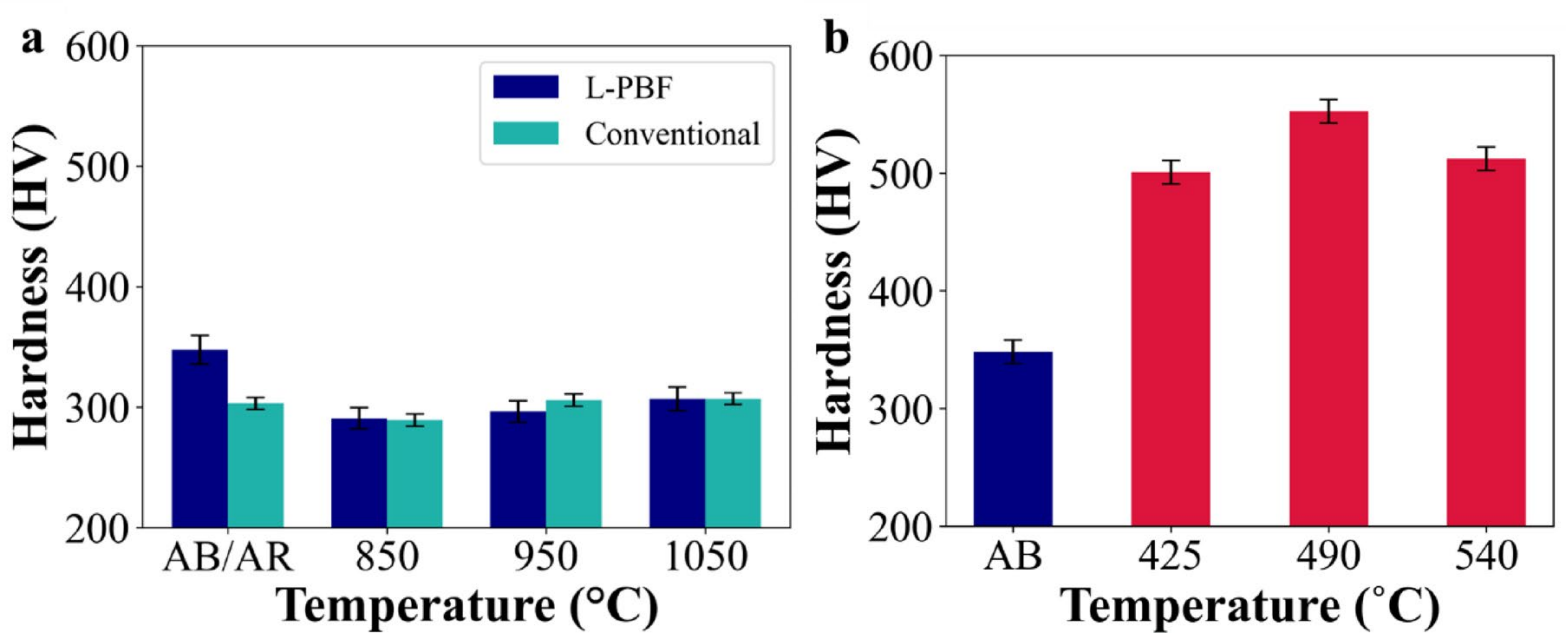
Vickers hardness of 18Ni(300) maraging steel at different heat-treatments: (**a**) as-built (AB)/As-received (AR) and solution annealed at 850 °C, 950 °C, and 1050 °C for 1 h (L-PBF and conventional), (**b**) solution annealed at 850 °C–1 h and aged at 425 °C, 490 °C, and 540 °C for 6 h (L-PBF).

The Vickers hardness of 18Ni(300) maraging steel under different heat-treatment conditions is shown in Fig. 12a. In as-built/as-received condition, L-PBF and conventional maraging steel exhibits a hardness of approximately 350 HV and 303 HV, respectively. Upon solution annealing at 850 °C for 1 h, the hardness of both L-PBF and conventional maraging steels significantly decreases to around 290 ± 10 HV. However, as the solution annealing temperature increases to 950 °C and 1050 °C for 1 h, there is an increase in hardness for both L-PBF and conventional steels, which is consistent with previous findings in the literature^11,17,28,65,66^. For example, Bai et al.^11^ and Venceslau et al.^28^ observed that the hardness of L-PBF maraging steel solution annealed at various temperatures gradually decreases, reaching a minimum, and then slightly increases as the temperature continues to rise, with the holding time maintained at one hour. L-PBF maraging steel maintains slightly higher hardness values across the temperature range compared to the conventional steel possibly due to finer grain sizes (Figs. 6 and 7).

The observed change in hardness following solution annealing at various temperatures can be attributed to changes in several key microstructural features: average grain size, the proportion of retained austenite, microstrain, and oxide particles. These factors are not only interconnected, but also vary in their influence on hardness under different conditions. At times, one or more of these factors may dominate the hardening effect, depending on the specific thermal history. This interplay ensures a dynamic response to heat treatment, where the dominant mechanism can shift, reflecting the complex nature of material behaviour under heat treatment^67–69^. The mechanisms through which these features influence hardness are outlined as follows: 1) Grain size and grain boundary effect: An important method for determining the relationship between microstructure and hardness/strength is the Hall-Petch (H-P) equation. In terms of hardness, the H–P equation can be expressed as follows (Eq. 3)^70,71^:

3 $$\:H={H}_{0}+k{d}^{-1/2}$$

In this equation, *H* represents the hardness, *d* represents the average grain size, and *H*_*0*_ and *k* represent the constants associated with hardness measurements, respectively. The Hall-Petch equation (Eq. 3) shows the relation between hardness and grain size. 2) Retained/reverted austenite: austenite (γ-phase) has an FCC crystal structure, while martensite (α-phase) has BCC crystal structure. Austenite, being an FCC phase, inherently possesses a higher number of slip systems compared to the BCC martensite phase typically desired in maraging steels for its high strength and hardness. These additional slip systems facilitate easier plastic deformation under applied stress, contributing to the softness of the material. Therefore, a decrease in the amount of retained austenite contributes to increased hardness. 3) Microstrain: Microstrain significantly influences the hardness of metals by impacting the crystal lattice and dislocation behaviour. High microstrain typically correlates with increased dislocation density, raising hardness through enhanced dislocation interaction that obstructs further movement. These interactions create a complex dislocation network, requiring greater force for deformation and thus directly enhancing hardness. Additionally, microstrain introduces lattice distortions that act as barriers to dislocation slip. This resistance to dislocation motion contributes to strain hardening, further increasing hardness. Moreover, microstrain around grain boundaries can amplify grain boundary-strengthening effects, particularly in a fine-grained structure. The overall impact is a pronounced increase in the material’s resistance to localised deformation, manifesting as increased hardness^67,68,72^. 4) Oxide particles: Oxide particles play a significant role in enhancing the hardness of maraging steels, particularly those produced by L-PBF, where finely dispersed oxides such as TiO_2_ and Al_2_O_3_ form due to the interaction of reactive elements with residual oxygen in the feedstock or build atmosphere. These oxides contribute to hardness primarily through oxide dispersion strengthening (ODS), as they act as obstacles to dislocation motion via the Orowan looping mechanism^31^. When dislocations encounter these particles, they are forced to bow and bypass them, increasing the stress required for continued deformation. This impedes plastic flow and contributes to strain hardening. In addition, oxide particles situated at or near grain boundaries exert a Zener pinning effect, which restricts grain boundary migration during solution annealing and helps to maintain a fine-grained microstructure. Oxide particles increase the resistance to deformation by impeding dislocation motion and inhibiting grain growth, both of which contribute to higher hardness in L-PBF maraging steels. Literature reports confirm that oxide dispersion in additively manufactured steels can significantly enhance hardness and mechanical strength, particularly following heat treatment^31–33,73^.

Based on the hardness results, solution annealing at 850 °C for 1 h is the most effective in reducing hardness, with the lowest measured value of 290 HV. This reduction results from the relief of residual stress and the decrease in dislocation density, which also lowers microstrain, as shown in Fig. 3. In contrast, hardness increases to 300 HV at 950 °C and 305 HV at 1050 °C. This increase is attributed to the presence of oxide particles, which exert a Zener pinning effect that restricts grain boundary migration during solution annealing. As a result, the growth of prior austenite grains is limited (Fig. 7), leading to the formation of finer martensite grains upon cooling (Fig. 8). This refined microstructure enhances hardness through the Hall–Petch effect. Additionally, the increased volume fraction of Ti- and Al-based oxide particles at higher temperatures (Table 4) contribute to dispersion strengthening by acting as obstacles to dislocation motion via the Orowan mechanism. Together, these effects account for the observed increase in hardness at higher annealing temperatures.

Figure 12b highlights the hardness evolution of L-PBF 18Ni(300) maraging steel solution annealed at 850 °C for 1 h and subsequently aged at 425 °C, 490 °C, and 540°Cfor 6 h. A significant increase in hardness is observed, with peak values reaching approximately 540 ± 5 HV at 490 °C. The hardness then slightly decreases to 512 ± 5 HV at 540 °C. Considering the hardness values of samples aged at various temperatures, aging at 490 °C for 6 h is determined to be the optimal procedure. According to the literature, maximum hardness, 540 ± 5 HV, can be achieved at 490 °C, which is in agreement with these works^37,38,74–78^.

**Fig. 13:**
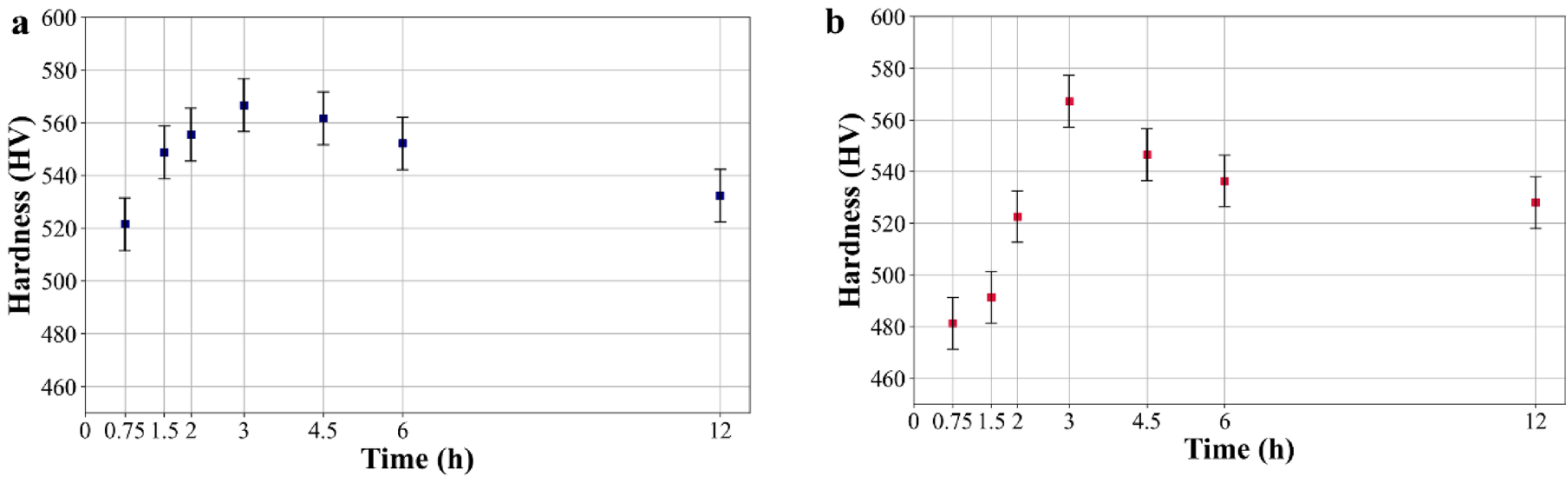
Hardness of aged 18Ni(300) maraging steel at 490 °C for various times: (**a**) L-PBF and (**b**) conventional.

Figure 13 illustrates the hardening curve of 18Ni(300) maraging steel aged at 490 °C, comparing L-PBF and conventional samples. Both L-PBF (Fig. 13a) and conventional (Fig. 13b) samples exhibit an initial increase in hardness, starting from approximately 290 ± 5 HV. After aging for 45 min, L-PBF samples reach a hardness of 521 ± 5 HV, while conventional samples only reach 481 ± HV, indicating a more pronounced initial hardening in L-PBF samples. Both sample types reach peak hardness values around 566 ± 5 HV for L-PBF and 567 ± 5 HV for conventional samples after 3 h of aging. After reaching the peak, the hardness decreases with further aging time, indicating the onset of over-aging, where precipitates coarsen and reduce the hardening effect. The observed slopes of the hardness curves indicate different aging kinetics between the two methods. Despite both reaching peak hardness at 3 h, L-PBF samples demonstrate a faster initial hardening process compared to conventional steel. This is confirmed by Johnson-Mehl-Avrami (JMA) equation (Eq. 4) that provides an excellent description of phase transformation theory^79–81^:

4 $$\:f=1-(-k{t}^{n})$$

In Eq. 4, *f* is the degree of transformation, *t* is the time after the transformation started; and *k* and *n* are two factors defining the transformation process, the rate constant and the Avrami exponent, respectively. This equation is derived from the fundamentals of age-hardening kinetics. By using the parameter *H*_*e*_, this equation can be applied to precipitation in 18Ni(300) maraging steel^80^:

5 $$\:{H}_{e}=\frac{{H}_{t}-{H}_{i}}{{H}_{max}-{H}_{i}}$$

In Eq. 5, *H*_*max*_ represents the maximum hardness, *H*_*i*_ and *H*_*t*_ represent the initial hardness and the hardness following precipitation for time *t*. It should be noted that the JMA equation is based on the assumption that a single phase can be formed from a matrix of another phase. However, during the precipitation of 18Ni(300) maraging steel, different precipitates, i.e., Ni_3_(Ti, Mo), Fe_2_Mo, and Fe_7_Mo_6_ may form simultaneously or overlap^14^. Even though, the JMA equation may not be the ideal method to describe each type of precipitate formation, aging curves obtained possess one main hardening peak which indicates one major phase controlling precipitation hardening. Additionally, the coexistence of different phases is complicated by the transformation between meta-stable precipitates. However, the JMA equation can be used to describe well the evolution of the total volume fraction of precipitates^79,80^. Hence, in this study the JMA equation has been applied to compare the age-hardening kinetics of L-PBF and Conventional 18Ni(300) maraging steel. As a result, in Eq. 4, *f* has been replaced by *H*_*e*_ (Eq. 3), and thus it is described as follows:

6 $$\:{H}_{e}=1-\text{e}\text{x}\text{p}(-{kt}^{n})$$

Based on the mathematical procedures used for Eqs. 5 and 6, it can be concluded that:

7 $$\:Ln(-ln(1-{H}_{e}\left)\right)=\:ln\left(k\right)\:+\:n\:ln\left(t\right)\:$$

**Fig. 14:**
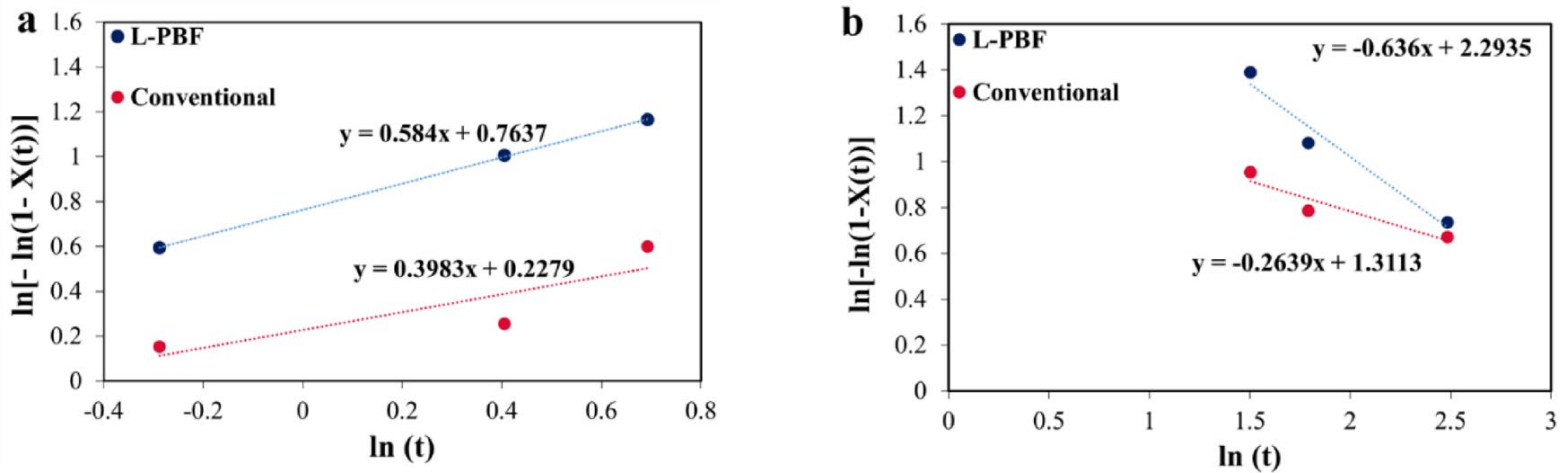
Plot for calculation of Avrami exponent (n) for precipitation (**a**) hardening and (**b**) coarsening.

Therefore, to obtain constant rate (*k*) and Avrami exponent (*n*) for precipitation reactions in 18Ni(300) maraging steel in the hardening and coarsening part, values of ln(-ln(1- H_e_)) versus ln(*t*) were plotted for aging at 490 °C as shown in Fig. 14. The linear regression of these data (lines with slope of *n* and intercept of *ln(k)*) results in the *n* values of 0.584 for L-PBF and 0.398 for conventional samples. Values for *k* are 2.15 and 1.25 for L-PBF and conventional 18Ni(300) maraging steel, respectively. Based on calculated Avrami rate constant and exponent, the L-PBF process exhibits faster precipitation and growth rate as indicated by the higher Avrami rate constant and a slightly more complex transformation mechanism as suggested by the higher *n* value.

The studies by Zhang et al.^82^Jägle et al.^27^and Kučerová et al.^66^ collectively investigate the subtle differences in precipitation hardening in maraging steel, examining both L-PBF and conventionally produced variants. Zhang’s work^82^ illustrates that Ni_3_(Ti, Al) precipitates form through heterogeneous nucleation at dislocations within a temperature range of 350–450 °C, leading to a swift increase in the material’s strength. From Jägle et al.^27^atom probe tomography reveals no significant differences in the size, density, chemical composition, and morphology of precipitates at peak hardening (480 °C for 6 h) between L-PBF and conventional steels, suggesting similar aging behaviours. Kučerová et al.^66^ delve into the impact of initial microstructure on precipitation hardening, comparing maraging steels in as-built L-PBF condition, solution annealed at 820 °C for 1 h, and 940 °C for 2 h, with conventionally manufactured maraging steel which was solution annealed at 820 °C for 1 h. As a result of their findings, it is evident that different initial microstructures influence their response to subsequent age-hardening, thereby affecting their mechanical properties after age-hardening.

In maraging steels, the microstructural defects such as dislocations and increased grain boundary areas serve as critical nucleation sites for the formation of precipitates, which are essential for hardening^14,27,82^. After solution annealing at 850 °C for 1 h, the L-PBF maraging steel retains a fine-grained structure with limited grain growth, resulting in a significantly higher grain boundary area compared to the conventionally processed steel, which exhibits a coarser, equiaxed grain structure. The increased grain boundary area in the L-PBF sample provides a greater number of potential nucleation sites for precipitation, which can enhance precipitation kinetics and promote a faster hardening response during ageing. Interestingly, despite the different initial microstructural conditions, both L-PBF and conventional maraging steels achieve the same level of peak hardness when subjected to aging at 490°Cfor 3 h. This convergence of peak harness values likely caused by the similar final volume fraction of strengthening precipitates. However, the routes to reaching this state differ fundamentally. In L-PBF samples, the abundance of dislocation networks and fine grain boundaries accelerates nucleation and promotes growth via pipe diffusion^83^leading to faster hardening kinetics. In contrast, the conventional steel, having equiaxed grain structure, exhibits slower precipitation but ultimately accumulates sufficient precipitate volume to reach similar peak hardness. The early advantage in L-PBF kinetics is thus balanced by more gradual but sustained transformation in the conventional steel. Nonetheless, more research is required to fundamentally understand the underlying mechanisms enabling this similarity, particularly given the distinct differences in their starting microstructures. This points to a complex interaction of factors influencing precipitate formation and growth, which are critical to the materials’ ultimate performance.

## Conclusions

- L-PBF 18Ni(300) maraging steel exhibited a fine, cellular microstructure with a high grain boundary area and dispersed oxide particles, which remained thermally stable during solution annealing.
- Grain growth was significantly limited in L-PBF samples, even at 1050 °C, likely due to the pinning effect of formed oxide particles. In contrast, conventional samples developed equiaxed grains at 850 °C and 950 °C, and coarser, irregular grains at 1050 °C.
- Despite these microstructural differences, both L-PBF and conventional samples achieved similar peak hardness after ageing. However, the L-PBF samples exhibited faster initial hardening, likely due to enhanced precipitation kinetics associated with their finer grain structure and greater number of nucleation sites.
- The application of the Johnson-Mehl-Avrami (JMA) theory demonstrated that the finer grain structure in L-PBF samples provided more nucleation sites for precipitates, speeding up the aging process.
- The similarity in peak hardness levels suggests that while the pathways to hardening differ, the end microstructural characteristics influencing hardness might converge under specific heat treatment conditions. However, more research is required to fundamentally understand the underlying mechanisms enabling this similarity.
- It is noted that conclusions regarding aging kinetics and precipitation behaviour are drawn primarily from indirect indicators, including hardness evolution, grain structure, and microstructural response, rather than direct observation of precipitates. This interpretive approach is acknowledged.

These findings offer important insights for the heat treatment optimisation of L-PBF-produced maraging steels. The findings indicate that oxide particle formation during solution annealing plays a key role in grain size control and contributes to improved mechanical performance. Understanding how microstructural features evolve with heat treatment allows for better design of post-processing strategies that improve performance and reliability. This is particularly significant for critical applications in aerospace, tooling, and defence industries, where additive manufacturing is increasingly used for high-strength, complex geometries.

## Data Availability

The datasets generated during and/or analysed during the current study are available from the corresponding author on reasonable request.
